# Impact of Viral Infections on Hematopoiesis: From Beneficial to Detrimental Effects on Bone Marrow Output

**DOI:** 10.3389/fimmu.2016.00364

**Published:** 2016-09-16

**Authors:** Maria Fernanda Pascutti, Martje N. Erkelens, Martijn A. Nolte

**Affiliations:** ^1^Landsteiner Laboratory, Department of Hematopoiesis, Sanquin, Academic Medical Centre, University of Amsterdam, Amsterdam, Netherlands

**Keywords:** hematopoiesis, HSC, HSPC, bone marrow, viral infection, antiviral, aplastic anemia, T cell

## Abstract

The ability of the bone marrow (BM) to generate copious amounts of blood cells required on a daily basis depends on a highly orchestrated process of proliferation and differentiation of hematopoietic stem and progenitor cells (HSPCs). This process can be rapidly adapted under stress conditions, such as infections, to meet the specific cellular needs of the immune response and the ensuing physiological changes. This requires a tight regulation in order to prevent either hematopoietic failure or transformation. Although adaptation to bacterial infections or systemic inflammation has been studied and reviewed in depth, specific alterations of hematopoiesis to viral infections have received less attention so far. Viruses constantly pose a significant health risk and demand an adequate, balanced response from our immune system, which also affects the BM. In fact, both the virus itself and the ensuing immune response can have a tremendous impact on the hematopoietic process. On one hand, this can be beneficial: it helps to boost the cellular response of the body to resolve the viral infection. But on the other hand, when the virus and the resulting antiviral response persist, the inflammatory feedback to the hematopoietic system will become chronic, which can be detrimental for a balanced BM output. Chronic viral infections frequently have clinical manifestations at the level of blood cell formation, and we summarize which viruses can lead to BM pathologies, like aplastic anemia, pancytopenia, hemophagocytic lymphohistiocytosis, lymphoproliferative disorders, and malignancies. Regarding the underlying mechanisms, we address specific effects of acute and chronic viral infections on blood cell production. As such, we distinguish four different levels in which this can occur: (1) direct viral infection of HSPCs, (2) viral recognition by HSPCs, (3) indirect effects on HSPCs by inflammatory mediators, and (4) the role of the BM microenvironment on hematopoiesis upon virus infection. In conclusion, this review provides a comprehensive overview on how viral infections can affect the formation of new blood cells, aiming to advance our understanding of the underlying cellular and molecular mechanisms to improve the treatment of BM failure in patients.

## Introduction

All blood cells develop from a small subset of the same progenitor cells in the bone marrow (BM) through a process called hematopoiesis. Hematopoietic stem cells (HSCs) give rise to red and white blood cells and platelets, and this hematopoietic process is tightly regulated to ensure both a balanced output of the different blood cells and a lifelong maintenance of self-renewing HSCs (1). The contribution of individual HSCs to blood cell production under steady state conditions is low, as this is mainly governed by multipotent progenitors with less or no self-renewing capacity (2). HSCs only occasionally give rise to new progenitors and are instead protected and nurtured by a complex microenvironment of resident hematopoietic and non-hematopoietic cells (3). This BM niche produces factors that maintain the quiescence, self-renewal, and survival of the HSCs. However, upon stress induced by cytotoxic damage, transplantation, inflammation, or infection, the pool of quiescent HSCs is activated and required to actively contribute to the hematopoietic process (4).

Several types of immune cells and inflammatory cytokines are involved in the skewing of hematopoiesis by bacterial infections or sterile inflammation (4, 5). The hematopoietic response to acute systemic bacterial infection, often referred to as “emergency granulopoiesis,” is characterized by systemic signs such as blood leukocytosis, neutrophilia, the emergence of immature neutrophils (clinically called left-shift), and increased production of myelomonocytic cells in BM (6). While the beneficial effect for the host to increase myeloid output to fight off a bacterial infection is evident, the desired BM output during a viral infection can be more complex. In this review, we give an overview of the impact of viral infections on hematopoiesis, and how the molecular feedback to the BM can contribute to improved viral clearance but also to either benign or malignant hematological disorders.

Viruses are small obligate intracellular parasites that require host cellular machinery for replication. That much said, there is an enormous variety of viruses, even of medically relevant viruses. Viruses that can infect humans range in size from 20 to 260 nm, are estimated to be of at least 30 different types and can cause pathologies, ranging from respiratory manifestations, enterocolitis, meningitis, encephalitis, hepatitis, and sexually transmitted diseases. Structurally, viruses are composed of a protein capsid that protects their genomic material and, in some cases, facilitates entry into the host cell. Some viruses can also be surrounded by a lipid bilayer – enveloped viruses, which also contain membrane glycoproteins that can interact with entry receptors on the surface of the host cells. The genome of viruses can be composed of DNA or RNA. RNA genomes can be coding, much like an mRNA molecule (positive-strand RNA) or have a complementary RNA molecule (negative-strand) that needs to be copied into a positive strand, which can then be translated by the cellular machinery. DNA viruses can have a single-stranded or double-stranded genome. The conformation of the genome can be linear or circular, and continuous or segmented. RNA genomes are limited by the inherent instability of RNA and usually contain fewer genes, with the smallest virus containing 3–4 genes. DNA genomes are generally bigger and might encode up to 200 genes. The size of the genome will impact the dependence of the virus on the cellular replication machinery and will also impact on how good the virus is in escaping immune responses. Bigger genomes are able to encode a broader range of sophisticated immune-evasion mechanisms (7).

A typical viral life cycle involves entry of the virus into the host cell, translation of viral proteins, replication of viral genome, assembly of viral particles, and final release of the mature virions into the extracellular milieu. All viruses have on their outside a receptor-binding protein. The receptors on the target cells invariably have another function but viruses have co-opted them for attachment to the cell. Once attached, the virus can enter the cell by fusion with the cell membrane, receptor-mediated endocytosis, or non-clathrin-mediated endocytosis. Eventually, the viral genome is released into the cytoplasm where it will be translated and replicated or reach the nucleus of the cell. Viruses code for strong signals to promote viral gene expression and other signals to repress expression of cellular genes. The expression of groups of viral genes is often carried out in critically timed phases with intermediate early genes coding regulatory proteins, early genes for genome replication proteins, and late viral genes for structural proteins. Once the viruses are assembled, the next step is release from the cell. Lytic viruses are released on lysis and death of the host cell. Non-lytic enveloped viruses bud from the cell surface. Viral infections can be acute, quickly resolved, or chronic, with some remaining in the host for its lifetime (7).

Viral infections start with local invasion, for example of an epithelial or mucosal barrier. Once the virus manages to overcome the early mechanical barriers, such as cilia, mucus, or skin integrity and infects a target cell, innate immune mechanisms come into play to contain the infection. As an example, during influenza virus infection, viral RNA present within infected cells is recognized by pathogen recognition receptors (PRRs), which leads to the secretion of type I interferons (IFNs), pro-inflammatory cytokines, eicosanoids, and chemokines. Type I IFNs stimulate the expression of hundreds of genes [IFN-stimulated genes (ISGs)] in neighboring cells, which induce an antiviral state. Pro-inflammatory cytokines and eicosanoids cause local and systemic inflammation, induce fever and anorexia, and instruct the adaptive immune response. Chemokines recruit additional immune cells, including neutrophils, monocytes, and natural killer cells to the airways. Virally infected epithelial cells become the target of NK cells. Monocytes and neutrophils help to clear infected dead cells, thus contributing to viral clearance (8). If the virus establishes infection despite these defenses, the ultimate clearance of the virus requires adaptive immunity, which relies on virus-specific antibodies and T cells. As an example, T cell responses against influenza comprise perforin/granzyme-induced lysis and tumor necrosis factor receptor family dependent apoptosis of infected cells and production of pro-inflammatory and regulatory mediators, such as IFNγ (9). In some cases, a strong antiviral response can even be more destructive than the virus itself and contribute to viral persistence (10–12).

## Adaptation of Hematopoiesis to Infections

Stress-induced hematopoiesis has most likely evolved to provide the body with the appropriate type(s) of blood cells to combat the invading pathogen. Signals originating from the infectious agent or the ensuing immune response can compel HSCs to change from a quiescent into a proliferative state and dictate the differentiation pathways of hematopoietic progenitors (13). For example, inflammation induced by immunization or LPS injection increases the production of granulocytes, macrophages, and dendritic cells but decreases the production of B cells (14, 15). Interestingly, the response of the hematopoietic system to bacterial products can also be influenced by non-immune cells, as BM stromal cells and endothelial cells can translate pathogenic information to lineage-specific differentiation through the production of pro-inflammatory cytokines or chemokines (16, 17). Macrophages also play an important role in the BM in regulating myelopoiesis, both during the steady state and upon inflammation, as was reviewed by McCabe and MacNamara (18). Inflammatory mediators, such as interferon-γ (IFNγ), skew the differentiation of hematopoietic stem and progenitor cells (HSPCs) toward monocytes at the expense of other lineages, as we reviewed previously (19). Boosting myelopoiesis during infections while inhibiting other lineages is probably related to the fact that myeloid cells are short lived, have little ability to expand in the periphery, and are rapidly consumed during acute bacterial infections. However, monocytes and granulocytes are not essential for the clearance of a viral infection, whereas lymphocytic cells, such as NK cells and T cells do have an important role in the immune response against viruses. Therefore, it is conceivable that significant differences exist in hematopoietic output upon an infection with a bacterial versus viral pathogen. In the coming section, we will discuss non-pathogenic and pathogenic outcomes of viral infections on BM output, and then, we will elaborate on four distinct effects by which viral infections can modulate the hematopoietic process.

## Pathogenic Outcome of Viral Infections

Numerous viral infections have been associated with BM failure or hyperproliferative syndromes. A summary of BM pathologies associated with human viral infections is outlined in Table 1, and a short description of terms and examples follows.

**Table 1:** **BM pathologies associated with human viral infections**.

Pathology	Virus	Comments	Reference
Pancytopenia	EBV	Self-resolving	(20)
	HCV	Afffected by medication	(21)
Aplastic anemia	Parvovirus B19	Driven by infection of erythroid progenitors	(22, 23)
	EBV, CMV, VZV, HHV, HIV, HAV, and HCV	Driven by a strong antiviral T cell response and ensuing cytokine production	(24, 25)
	Dengue	Mechanism unknown	(26)
HLH	CMV	Driven by the ensuing antiviral immune response rather than the virus itself	(27, 28)
	Parvovirus B19		(22)
	Dengue		(29, 30)
	HAV		(31)
	HIV (acute)		(32)
Lymphoproliferative disorders and malignancies	EBV	Infectious mononucleosis and chronic active EBV disease	(20)
	HCV	Acute myeloid leukemia, primary myelodysplastic syndrome	(21)

*In this table, we summarize the viruses that can contribute to particular type of pathology in human BM*.

*Pancytopenia* is a deficiency of all three blood cell types: red blood cells (anemia), white blood cells (leukopenia), and platelets (thrombocytopenia). In rare cases, it can appear as a direct self-resolving consequence of viral infection, such as that observed during EBV-associated infectious mononucleosis (20), but in most cases, it is secondary to other hematological disorders, such as aplastic anemia or hemophagocytic lymphohistiocytosis (HLH). HCV-infected patients are also prone to developing peripheral cytopenias, which has been proposed to be a multifactorial process also influenced by antiviral medication, such as Ribavirin (21).

*Aplastic anemia* is a BM failure condition where the BM contains very few hematopoietic cells and consists mainly of fat. Because of defective hematopoiesis, aplastic anemia results in pancytopenia. Viral infections associated with aplastic anemia include parvovirus B19 (22, 23), Epstein–Barr virus (EBV), cytomegalovirus (CMV), varicella-zoster virus (VZV), human herpes virus 6 (HHV-6), human immunodeficiency virus (HIV), hepatitis A and C viruses (HAV and HCV), and dengue (21, 24). Parvovirus B19 is highly tropic to human BM and replicates only in erythroid progenitor cells. In individuals with underlying hemolytic disorders, infection with parvovirus B19 is the primary cause of transient aplastic crisis. In immunocompromised patients, persistent B19 infection may develop and lead to pure red cell aplasia and chronic anemia (22). It has been proposed that, in most acquired cases, the hematopoietic tissue is the target of oligoclonal CD8^+^ T cells, which secrete IFNγ and TNFα and cause hematopoietic cell death (19, 25). Alternatively, continuous production of these pro-inflammatory cytokines can also exhaust the HSC compartment and thereby lead to aplastic anemia (5, 19, 33).

*Hemophagocytic lymphohistiocytosis* is a rare hyperinflammatory syndrome that is characterized by an uncontrolled activation of macrophages and lymphocytes and a life-threatening cytokine storm, accompanied by pancytopenia, among other complications. Primary HLH is caused by mutations in genes that regulate granule-dependent cytotoxicity of cytotoxic T cells and NK cells. Secondary HLH has infectious and non-infectious triggers. Among the infectious triggers, viral infection is the most frequent, either as a primary infection in healthy people or after reactivation in immunosuppressed patients. Herpes viruses, such as EBV and CMV account for 62% of reported viral cases of HLH in adults (34). Five murine models of genetic HLH have been established to study the pathogenesis of HLH. All of them display the same disease manifestations as humans and require a viral trigger to develop HLH (35). Murine CMV infection models have highlighted that pathologic mechanisms may be different in primary and secondary HLH (27). Pathogenesis of primary HLH is associated to hyper-activated CD8^+^ T cells, producing large amounts of IFNγ (28), while CD8^+^ T cells seemed dispensable, and IFNγ had more of a regulatory than a pathogenic role during secondary HLH (27). Other viruses, such as Dengue, have also been associated to this pathology (29, 30), although causative relationships are still missing. All in all, the systemic and BM-associated pathology, i.e., pancytopenia, in HLH seem to arise due to excessive inflammation, not by direct effect of the viral infections. However, the question remains as to why certain viruses can trigger such excessive responses and to what extent this is related to the genetic or immunological makeup of the host.

Finally, some viral infections, such as EBV, may lead to lymphoproliferative disorders and/or lymphoid malignancies. EBV-driven B cell lymphoproliferative disorders have been extensively studied and arise from latently infected B cells. Of all the different manifestations, which can affect different lymphoid organs and peripheral tissues, BM involvement has been described during infectious mononucleosis and chronic active EBV disease. Reported BM involvement included pancytopenia, as mentioned before, and detection of proliferating transformed cells (20). Patients with HCV infection develop a number of hematologic disorders, with benign and malignant B cell proliferations being the most common (21). HCV can infect, but not replicate, in B cells, and B cell proliferation in HCV-infected patients seems to result from chronic antigenic stimulation (21). All in all, BM involvement associated with lymphoproliferative disorders and malignancies seem to arise through, yet uncharacterized, indirect mechanisms and not by direct transformation of HSPCs.

In general, most reports regarding the effect of viral infections on BM output refer to pathologic conditions where hematopoiesis is seriously perturbed. It is likely that many acute viral infections induce transient alterations on the hematopoietic process, through the action of mediators such as type I IFNs, TNF, and lymphotoxin (LT), as has been described in mice models of lymphocytic choriomeningitis virus (LCMV) (36) and Influenza infections (37). In the case of LCMV, transient BM aplasia is dependent on type I IFNs (36). In influenza-infected mice, B cell precursors transiently decreased in numbers in a TNF- and LT-dependent manner (37). These effects are most likely overlooked in many human acute viral infections because of their subclinical nature.

Although the particular pathogenic mechanisms underlying all these BM manifestations are not fully understood, two observations seem to stand out from the data. First, many different viruses give rise to the same pathological outcome, which suggests that common underlying mechanisms either of virological or immunological origin might be responsible, and indeed, the immune response plays a major role in the pathogenesis of aplastic anemia and pancytopenia. Second, the fact that a certain virus can lead to different pathological manifestations in different individuals points to a genetic basis for aberrant immune activation in BM failure, as suggested before (19, 25). In general, mechanistic studies in animal models are scarce. Dissection of common and pathogen-specific mechanisms could greatly improve therapy and management of affected patients.

As a general conclusion, many types of viruses can affect hematopoiesis and, in this section, we have described examples of acute (Parvovirus B19, dengue) and chronic (CMV, HIV), systemic (HIV), and localized (Influenza) infections, which directly or indirectly, transiently or more permanently, affect the hematopoietic process. In the following sections, we will elaborate on well characterized and also proposed mechanisms behind these processes.

## Mechanisms of Viral Interference with Hematopoiesis

**Figure 1:**
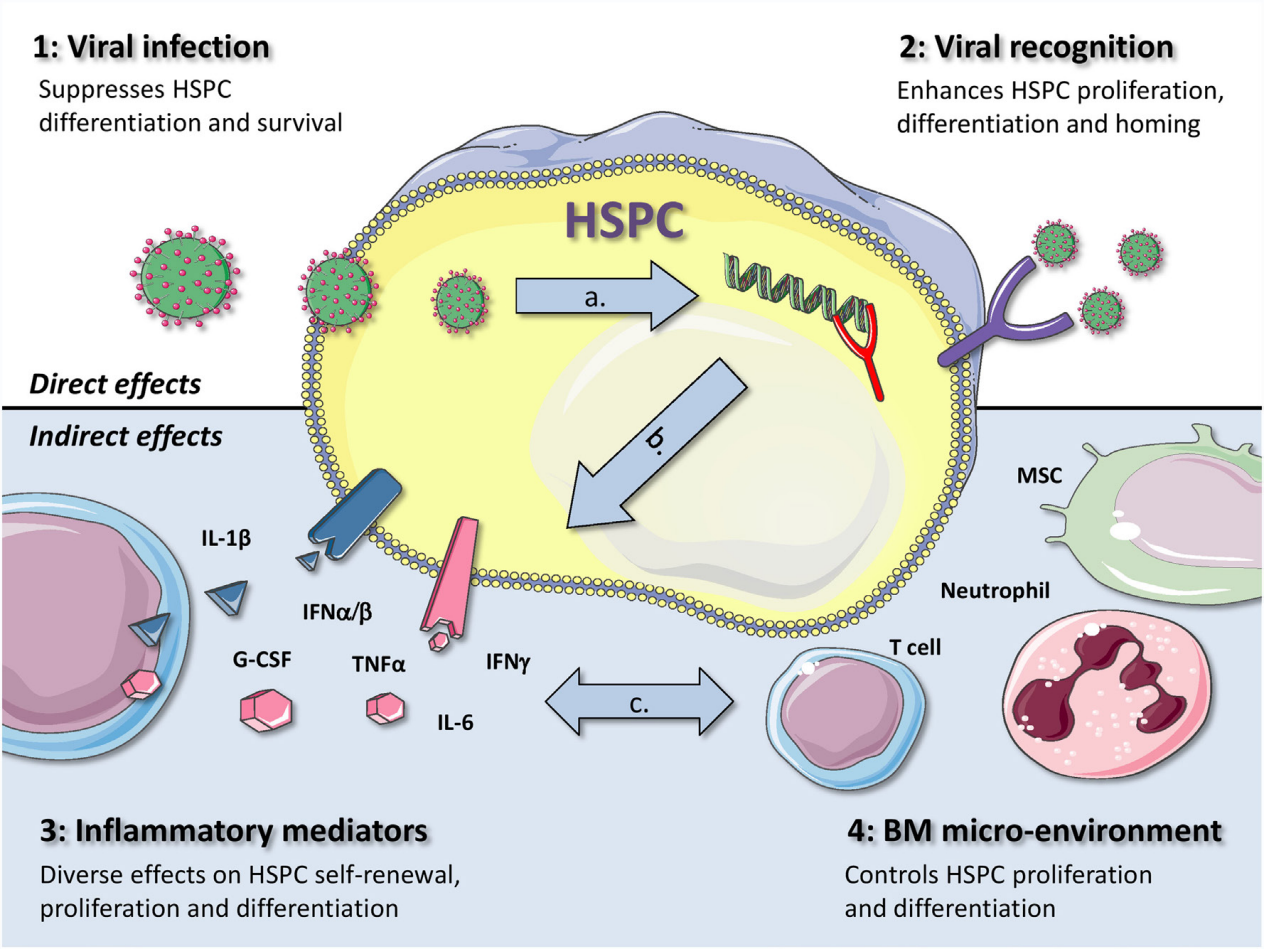
**Graphical representation of four different mechanisms by which viral infections can influence the function of HSPCs**. The first two mechanisms act *via* direct effects on HSPCs: (1) direct viral infection or (2) viral recognition by HSPCs. The other two mechanisms are indirectly: (3) *via* inflammatory mediators or (4) through changes in the BM microenvironment. During a viral infection, more than one of these mechanisms contribute to alterations in hematopoiesis, as they are also likely to influence each other, as indicated by the gray arrows. This is exemplified by the fact that **(A)** when a virus infects an HSPC, it is generally also recognized through intracellular PRRs, **(B)** recognition of viral infections by HSPCs usually also leads to the production of pro-inflammatory cytokines, such as type I IFNs, and **(C)** production of IFNγ by virus-specific T cells can directly affect HSPCs but can also induce IL-6 production by MSCs, thereby enhancing myeloid differentiation. Better understanding of the complex interactions between these different mechanisms will be important to adequately treat or prevent anemia and BM failure in patients with viral infections. (The illustrations used to generate this figure are gratefully obtained from the Powerpoint Image Bank of Servier Medical Art).

Both viruses and immune responses directed toward them have an impact on hematopoiesis. In an elegant review, King and Goodell previously categorized four different mechanisms by which infections in general can influence HSC biology (4). The first two mechanisms act *via* direct effects on HSCs: (1) direct infection or (2) direct recognition of a pathogen. The other two mechanisms are indirect: (3) either *via* pro-inflammatory cytokines released by other cells or (4) through changes in the BM microenvironment. These four scenarios are not mutually exclusive and can even enhance or attenuate each other. In the coming sections, we will use this subdivision to describe how viral infections can affect BM output in general and the function of HSPCs in particular (Figure 1).

### Direct Viral Infection of HSPCs

It has been described that a number of viruses including, CMV, Hepatitis C virus (HCV), and human herpes viruses can directly infect HSPCs (38–41). For some of these viruses, it has been documented that they can suppress hematopoiesis after direct infection of HSPCs. For example, Simmons et al. demonstrated that CMV infection can mediate myelosuppression *in vitro* (42). Furthermore, human herpes viruses 7 (HHV7) has the potential to infect HSPCs as well as impair HSPC survival and proliferation, presumably *via* lysis, or induced cell death (41). Parvovirus B19, the only known human pathogenic parvovirus, has a selective tropism for the erythroid lineage in the BM, where productive infection induces a block in erythropoiesis that can be manifested as a transient or persistent erythroid aplasia (43).

Moreover, the consequence of direct viral infection of HSPCs, such as changes in the expression of intracellular factors e.g., microRNAs, may impact hematopoiesis as well. It was shown that retroviruses such as HIV and HTLV target microRNAs to manipulate key cellular pathways, which can result in the development of hematopoietic malignancies such as B cell and Hodgkin’s lymphomas (44, 45). Furthermore, it was observed that both HIV and SIV can induce large hematopoietic defects. Prost et al. demonstrated that the inhibitory effects on hematopoiesis of SIV depend entirely on the presence of the viral protein Nef. They showed that SIV affected HSPCs by downregulating STAT5a and STAT5b *via* Nef *in vivo* and demonstrated that SIV strongly downregulates early hematopoiesis in this manner (46).

Overall, direct viral infection in HSPCs has been shown to reduce the hematopoietic output. However, the exact underlying mechanisms after direct virus infections in HSPCs have not been fully elucidated. It is conceivable that the halt of translation in host cells (or “shut off”) that occurs in many cells following viral infection contributes to this process, though the impact of this antiviral mechanism on HSPC function is not clear.

It has been proposed that direct infection of HSCs does not commonly occur, as they usually reside in protected BM niches (4). Because of this protected microenvironment, quiescent HSCs are thought to be resistant against bacterial infections (47). In fact, all the evidence described in the preceding paragraphs does not distinguish between infection of HSCs or downstream progenitors. It remains to be determined whether there are any differences in virus susceptibility between these cell types. It is likely that quiescent HSCs are less likely to become infected with a virus compared to their non-quiescent counterparts, and evidence supporting this notion is described in the following section.

### Role of Direct Recognition of Viral PAMPs in Stress-Induced Hematopoiesis

Pathogen recognition receptors are receptors that detect pathogen-associated molecular patterns (PAMPs) within the body (48, 49). PRRs include toll-like receptors, retinoic acid-inducible gene 1 (RIG-I)-like receptors (RLRs), and cyclic guanosine monophosphate-adenosine monophosphate (cGAMP) synthase (cGAS) (50). Four TLR members seem to play a critical role in recognition of viral nucleic acids: TLR3 recognizes dsRNA (dsRNA constitutes the genome of one class of viruses but is also generated during the life cycle of many viruses), TLR7 and 8 recognize single-stranded RNA (ssRNA), and TLR9 responds to dsDNA viruses recognizing non-methylated viral CpG-containing DNA. Although the majority of TLRs sense pathogen components on the cell surface, TLR3, TLR7, TLR8, and TLR9 sense nucleic acids in endosomal compartments. Other TLRs are also involved in viral recognition; TLR2 and TLR4 were shown to detect viral components such as envelope glycoproteins (51) and other components of viruses such as HIV, HBV, vaccinia virus (VV), and Dengue (52–55).

Interestingly, several intracellular and extracellular TLRs have been found on HSCs (13). It was shown both *in vitro* and *in vivo* that direct TLR ligation triggered cell cycle entry in quiescent HSCs, bringing them into a proliferative state (14, 56, 57). This indicates an active role for HSCs in immune sensing, and the modulation of early hematopoiesis during infection. De Luca et al. reported that human HSCs and lineage-committed progenitors express TLR3 (58), whereas Sioud et al. showed that HSPCs particularly express TLR7 and TLR8 (57). These different observations could be explained by different sources of cells that were used, as De Luca et al. isolated HSPCs from cord blood, whereas Sioud et al. isolated these cells from BM of healthy donors. Although side-by-side validation is lacking, differential TLR expression in fetal vs. adult HSCs suggests that these cells may be equipped to detect and respond to different viral infections. Furthermore, HSPCs also express TLR2 (14, 59). Finally, exposure of murine HSPCs to TLR ligands has also been shown to modulate their chemokine receptor expression (6) suggesting that TLR triggering may even regulate their migratory and homing capacities. In line with this, a comparison of TLR expression patterns between BM resident and mobilized HSPC could reveal interesting differences as to their response to viral infection in terms of lineage commitment and adaptation to demand. It might be that mobilized HSPCs in tissues have an increased surveillance capacity than those in the BM, but this remains to be demonstrated.

While signaling through TLR7 and TLR8 resulted in HSPC differentiation along the myeloid lineage (57), in the presence of ligands for TLR9, the *in vitro* generation of DCs dramatically decreased in favor of the production of macrophages (60). Correspondingly, it was demonstrated that the potential of myeloid progenitors to produce DCs was reduced upon TLR9 ligation (61). However, lymphoid progenitors from mice with a herpes virus infection were biased toward DC differentiation, which was dependent on TLR9, and that treatment of uninfected mice with the TLR9 ligand CpG ODN resulted in an increased DC generation (61). These findings indicate that TLR9 triggering differentially affects the generation of myeloid vs. lymphoid lineage-derived DCs. Furthermore, in the setting of acute leukemia, Dorantes-Acosta et al. found that leukemic HSPCs respond differently to various TLR ligands (62). Their findings suggested that B cell development is only marginally influenced by infectious agents, whereas they observed an increased production of myeloid and NK cell types in response to infections and disease-associated cell damage. For example, they observed an increase in the development of mature CD56^+^CD11c^+^ NK cells after stimulation of TLR8 and TLR9 in chronic acute leukemia-derived BM cells (62).

Besides affecting lineage commitment, viral sensing might also directly affect HSPC survival. Ligation of the RLRs RIG-I/MDA-5, but not TRL3, by poly I:C triggered apoptosis of human CD34^+^ cells, through a type I IFN-independent, caspase-dependent mechanism (63). It has, in fact, been shown that RLRs can trigger a dual response in virus-infected cells, which are independent and both contribute to viral control (64, 65). RLR-signaling activates IRF-3, which then migrates to the nucleus and binds to the IFN-stimulated response elements (ISRE) in the promoters of the target genes to induce type I IFN expression (64). In parallel, IRF3 can also activate the RLR-induced IRF-3-mediated pathway of apoptosis (RIPA) (65). It has long been reported that not every infected cell produces type I IFN (66, 67); there is heterogeneity in the type I IFN production within populations of virus-infected cells, which may be linked to expression levels of RIG-I signaling components or heterogeneous chromatin status of IFNβ genomic locus (67). It might be that the quiescent HSCs respond differently to RLR triggering than downstream progenitors. It is to be expected, given the relevance of maintaining the HSC pool for life, that quiescent HSC are more prone to inducing type I IFN production, which is followed by activation of numerous antiviral mechanisms, instead of inducing the RIPA pathway that kills the cells. There is potentially less biological cost in inducing RIPA in multipotent progenitors to limit viral replication, because these cells can be replaced by long-term HSCs later on. In line with this, a comprehensive proteomics analysis (68) revealed differences between HSPCs (Lineage^-^Sca-1^+^c-kit^+^ cells) and myeloid progenitors (Lineage^−^ Sca-1^−^c-kit^+^ cells). Strikingly, 2 ′–5′ oligoadenylate synthetase 3 (Oas3), RIG-I, and Ifit1, three different cytoplasmic sensors for viral RNA, were strongly upregulated in the multipotent state as well as a number of antiviral interferon-stimulated proteins. These results suggest that, compared to downstream progenitors, HSPCs might actually be more prone to activating the IFN antiviral response, although differences between quiescent HSC and multipotent progenitors remain to be shown. In fact, HSPCs are poorly permissive to both retroviral- and lentiviral-based gene transfer. Lentivirus has the advantage that they do not require cycling cells to integrate their genome and could potentially be more suitable for transformation of quiescent HSCs. Nevertheless, genetic manipulation of HSPCs still requires the use of multiple hits of high vector doses and prolonged *ex vivo* culture, suggesting that permissiveness is not only dependent on cell-cycle but also on the activation of multiple innate immune sensors and restriction factors that limit viral infection (69).

Altogether, it is clear that HSPCs can respond to viral infections through direct recognition of several viral PAMPs, and that the activation of different PRRs can result in different biological outcomes, ranging from changes in chemokine receptor expression and lineage-specification to induction of apoptosis.

### Indirect Effects of Viral PRR Triggering on Hematopoiesis

In response to PRR ligation by viruses, immune and non-immune cells produce a number of cytokines and chemokines. These include IL-1β, IL-6, IL-8, IL-10, TNFα, GM-CSF, TGFβ, CCL3 (MIP-1α), CCL4 (MIP-1β), CXCL10 (IP-10), and type I IFNs. Different viruses induce slightly different patterns that depend on the interaction of the particular viral PAMP with the specific PRR and the cell type involved (70, 71). In general, pro-inflammatory cytokines like IL-1β, IL-6, and TNFα together with type I IFN production are common to all viral, and many bacterial, infections (70). Many viruses that persist, such as EBV, HIV, and HTLV-I, signal innate cells such as dendritic cells, NK cells, and macrophages to produce anti-inflammatory molecules such as IL-10 and TGFβ (70, 71).

High levels of IFNα directly induces HSCs to exit quiescence and transiently proliferate *in vivo* (72, 73). Since most cell types stop proliferating in response to IFNα, the wiring of this signaling pathway must be fundamentally different in HSCs (74). According to Pietras et al. (75), type I IFN-driven HSC proliferation is a transient event resulting from a brief relaxation of quiescence-enforcing mechanisms in response to acute type I IFN exposure, which occurs exclusively *in vivo*. This proliferative burst fails to exhaust the HSC pool, which rapidly returns to quiescence in response to chronic type I IFN exposure, achieved by repeated polyI:C administration, because of the presence of intrinsic regulatory mechanisms. Type I IFN-exposed HSCs with re-established quiescence are not fully functional but are also largely protected from the killing effects of IFNs unless forced back into the cell cycle due to culture, transplantation, or myeloablative treatment, at which point they activate a p53-dependent proapoptotic gene program (75). These ideas are further supported by independent findings showing that, when genes that normally suppress IFN signaling are disrupted, mice have increased levels of IFN signaling, and their HSC populations are depleted over time (73, 76, 77). In chimeric mice transplanted with both wild-type HSCs and IFNα-receptor-deficient (IFNαR^−/−^) HSCs and later exposed to IFNα, only the IFNαR^−/−^ HSCs survived (72). In fact, exit from quiescence, induced by polyI:C or other stimuli, leads to generation of DNA damage that activates a DNA damage response in HSCs. Repeated activation of HSCs out of their dormant state provoked their attrition, and this was exacerbated in mice with a defect in DNA repair, suggesting that inefficient repair of replicative DNA damage may result in HSC depletion (78). Additionally, one single dose of IFNα or polyI:C induced an increase in the production of megakaryocyte-related proteins by acting on a stem-cell-like megakaryocyte progenitor that is contained within the phenotypically characterized HSC pool. Again, chronic exposure to polyI:C triggered exhaustion of these stem-cell-like megakaryocyte progenitors and a delayed repletion of platelet counts (79). Recent work also proposes that chronic exposure to IFNα drives medullar lymphopoiesis toward T-cell differentiation, while impairing the generation of B, NK, myeloid cells, erythrocytes, and platelets (80). Collectively, these findings suggest that BM aplasia associated with chronic exposure to type I IFN could arise from a depletion or loss of function of progenitors, together with enforced quiescence of HSCs, which become less functional. Moreover, if subsequent inflammation or infections force these quiescent HSC chronically exposed to type I IFNs back into cell cycle or if control mechanisms such as those regulating IFN signaling or DNA damage repair are deficient in certain susceptible individuals, this might lead to rapid depletion of the HSC quiescent pool and ensuing BM failure.

After the first wave of type I IFNs produced by infected cells, type II IFN (IFNγ), produced by stimulated T cells and NK cells, also contributes to the impairment of HSC self-renewal (81, 82). IFNγ triggering on HSPCs enhances monocyte formation, but this is at the expense of the production of neutrophilic (83) and eosinophilic granulocytes (84), B cells (85, 86), and erythrocytes (87, 88). These data illustrate that IFNγ has a wide-ranging effect on the hematopoietic process in the BM (19). It seems clear that type I and type II IFNs have unique features, with type I IFNs leading to megakaryopoiesis on the short term and quiescence and T-cell-biased lymphopoiesis upon chronic exposure, while IFNγ favors myelopoiesis at the expense of other branches of differentiation. This might link the temporal expression of the two types of IFN to the changing needs of the immune response as it progresses from initial innate control to adaptive mechanisms.

As mentioned before, PRR stimulation by viruses can also lead to the production of pro-inflammatory cytokines and chemokines, which can in turn affect the proliferation and differentiation of HSCs. Several cytokines such as IL-1β, IL-6, TNFα, TGFβ, M-CSF, and GM-CSF have been identified to have the capacity of regulating the proliferation and differentiation of HSCs as reviewed by Mirantes et al. (33). Overall, these results indicate that cytokines can instruct the differentiation and proliferation of HSCs, with BM output being a complex integration of signals from the microenvironment. As with prolonged type I IFN exposure, overproduction of inflammatory cytokines is often associated with hematopoietic failure such as chronic inflammatory diseases and hematopoietic malignancies (33), but the individual contribution of each player, and their potential synergies and antagonisms, remains to be determined. Finally, apart from inflammatory cytokines, costimulatory molecules may also play a significant role in altering hematopoiesis during viral infection (5). One example is the interaction between CD27 and CD70, which is important in the control of T cell immunity against Influenza (89) and CMV (90). This is of interest for the BM, as we previously showed that CD27-triggering on HSPCs may serve as a negative feedback mechanism that can regulate hematopoiesis during inflammatory conditions (91). This provides another layer of complexity by which immune activation can modulate the BM output upon viral infection.

### Role of the BM Microenvironment on Hematopoiesis upon Virus Infection

As described for sterile inflammation and bacterial infections, viral infections may also affect hematopoieisis indirectly *via* the BM microenvironment. Apart from hematopoietic cells, the BM also contains non-hematopoietic components, such as osteoblasts, mesenchymal stromal cells (MSCs), adipocytes, perivascular cells, endothelial cells, and non-myelinating Schwann cells. It has long been recognized that non-hematopoietic stromal cells in the BM are capable of supporting long-term hematopoiesis *in vivo*, and that the integrity of several populations of cells is crucial for the long-term maintenance of the quiescent HSC pool (92, 93).

Infection of mice with different strains of LCMV leads to transient type-I IFN-dependent BM aplasia that reaches a minimal BM cellularity within 3 days and recovers within 10 days after infection, coinciding with viral clearance. Although the mechanisms have not been clearly dissected, LCMV can infect both stromal cells and megakaryocytic and myelocytic precursors (36). It is likely that the aplasia is a result of apoptosis of the hematopoietic progenitors, which might be modulated by type I IFN signaling, as described above. However, the effects of viral infection on the stromal cells have not been dissected. It would be very interesting to address whether these cells die from apoptosis or respond by secreting cytokines that can affect hematopoiesis. If different populations of stromal cells are depleted and the hematopoietic niches are destroyed, the mechanisms by which these niches are reconstituted after viral clearance are still unknown. In this line, an important role for stromal cells in the hematopoietic stress-induced response during LCMV infection has been demonstrated by Schürch et al. (94). They described that IFNγ produced by CD8 T cells during the adaptive response to LCMV can also affect HSCs in an indirect manner by inducing IL-6 production in non-hematopoietic stromal cells (94), which in turn enhances myelopoiesis by downregulating the expression of Runx-1 and Cebpα in hematopoietic progenitor cells. Importantly, this increase in myelopoiesis was not only seen upon LCMV infection but also upon infection with vesicular stomatitis virus (VSV) or VV, suggesting that it is a general response to viral infection (94). These observations indicate that IFNγ not only acts directly on HSPCs, as described above, but also in an indirect manner *via* non-hematopoietic BM cells.

Mesenchymal stromal cells have also been reported to be affected by HIV, by a mechanism involving Tat and Nef (95). MSCs chronically treated with Tat and/or Nef reduced their proliferative activity, underwent early senescence, and showed decreased potential for osteoblastic differentiation, which could explain why HIV infected individuals present a higher prevalence of osteopenia/osteoporosis. Although no direct link was made, loss of the BM MSC functionality may have consequences on hematopoiesis.

Additionally, BM MSCs have been proposed to be a natural reservoir for human CMV (96). A recent retrospective study has shown that the CMV status of both donor and host are relevant for the overall survival of patients receiving allogeneic stem cell transplantation (HSCT) (97). As expected, CMV-negative recipients benefit from receiving HSCT from a CMV-negative donor, since the virus transmitted from a seropositive donor into an immunosuppressed seronegative recipient can have devastating effects. More intriguingly, CMV-positive recipients have an increased overall survival after receiving HSCT from a CMV-positive donor after myeloablative conditioning but not after reduced-intensity conditioning. Interestingly, there were differences in the causes of death between patients with CMV-seropositive or CMV-seronegative donors. Patients who received grafts from CMV-seronegative donors were more likely to die from viral infection given as the only cause of death or where a viral pathogen was included as a part of a mixed infection with different organisms. This effect was abrogated by T-cell depletion (97), which suggests that memory T cells from the donor can help control viral infections in situations of more aggressive conditioning. Since recipient MSCs are not depleted during either of the conditioning regimes (98), and they can harbor CMV, it would be interesting to study the interaction between donor CMV-specific memory T cells and host CMV-infected stroma, and how this interaction is modulated by the conditioning treatment.

Another virus that has a significant impact on the BM microenvironment in mice is the type B coxsackievirus (CVB). Althof et al. observed that after 3–4 days of infection, the femoral BM stroma was largely destroyed. While granulocyte and macrophage progenitors showed a relatively normal proliferative capacity, there was a marked decrease in colony-forming capacity in both erythroid and lymphoid progenitors, indicating a differential impact on the various HSPC subsets (99). Production of type I IFNs was shown to contribute to the development of lymphopenia upon CVB infection (99), which could also further contribute to the hematopoietic defects of the other hematopoietic lineages.

Although the contribution of the BM microenvironment to stress-hematopoiesis can be mediated through direct recognition of the pathogen and/or the ensuing production of inflammatory mediators, it may also simply be driven by the absence of particular cell types. Scumpia et al. observed that HSPC expansion upon bacterial infection can occur even without sensing of the bacterial PAMPs (using MyD88^−/−^ TRIF^−/−^ mice). They suggest that reduction in BM cellularity alone is sufficient to induce HSPC expansion, possibly mediated by supporting stromal cells that can provide the necessary signals for HSPC expansion within the BM space left void following infection or chemotherapeutic BM ablation (100). Similarly, it has been suggested that granulopoiesis can be driven by the number of neutrophils present in the BM. At low neutrophil numbers, macrophages and DCs produce IL-23, which stimulates IL-17 production by T cells, which in turn increases granulopoiesis *via* G-CSF. On the other hand, at high neutrophil numbers, the production of IL-23 in DCs and macrophages is inhibited by the phagocytosis of apoptotic neutrophils, resulting in a negative feedback loop (101). Large numbers of neutrophils are predominantly important in fungal and bacterial infections, and whether similar interactions are at play in viral infections is currently unknown. However, it is an interesting concept that the sheer presence or absence of particular cell types in the BM can also contribute to the virally induced hematopoietic stress response.

Overall, the link between the BM microenvironment and hematopoiesis is not easy to define. Multiple pathways can play a role in the regulation of hematopoiesis *via* the BM microenvironment, and these pathways may further complement each other. Yet, understanding the underlying cellular and molecular mechanisms by which the BM responds to viral infections may help us to counteract the ensuing pathogenic consequences, which were discussed at the beginning of this review.

## Conclusion and Future Directions

Viral infections can cause direct and indirect damage to HSPCs and the surrounding tissue. Direct pathogenic effects depend on viral tropism and viral cycle, and there are a few examples of direct infection of HSPCs that lead to altered BM output, e.g., parvovirus B19. But the complex interactions between viruses, HSPCs, and the BM microenvironment are underappreciated at the moment. One such case is CMV, which can infect both stroma and HSPC, with the end result of chronic latent infection and no overt BM pathology. Acute viral infections usually cause transient aplasia, partly related to the effect of type I IFNs, and to direct viral infection, in which both HSPCs and stromal cells are depleted. The mechanisms involved in repopulation of the BM by HSCs are well known. However, very little is known about how MSCs and other stromal cells recover from acute damage and by which mechanisms the different BM niches are reconstituted after resolution of infection. Insights into these processes could help understand BM repair in other situations, such as after radiation or chemotherapy. This information has direct translatable potential into the clinic, as enhanced niche reconstitution could be highly beneficial after HSC transplantation. Finally, a link between direct viral infection of HSPCs and transformation has not been found. Instead, some hyperproliferative syndromes arise after infection of mature B cells, and BM involvement appears as a secondary condition. Indirect damage arising from acute or chronic viral infection has been frequently attributed to the antiviral immune response, with IFNγ and CD8^+^ T cells playing a major role. However, BM pathologies are rare events and often associated to alterations in gene regulation of cytokines, effector, and MHC molecules, which suggest a genetic basis for aberrant T cell activation in BM failure [reviewed in Ref. (19, 25)].

Furthermore, one final interesting aspect that deserves exploring is the concept of the BM as a site of immune privilege. The BM contains a high proportion of regulatory T cells (Tregs), which have been proposed to protect the HSC niche (102). Although this concept was tested under conditions of allogeneic HSC transplant, there are no examples of control of antiviral responses by BM Tregs. It would be very interesting to dissect how Tregs shape the interactions between viruses, HSPCs, and stroma, and how this affects viral clearance, skewing of hematopoiesis, and niche regeneration.

Overall, hematopoiesis is a very flexible process that quickly adapts to the needs of the host, providing an adjusted cellular output to fight off a certain pathogen. While considerable insight has been gathered regarding the regulation of BM output by general inflammatory processes and bacterial infections, less is known about the specific regulation of hematopoiesis by viral infections. Some of the previously described mechanisms also apply to this situation. Virus-specific T cells produce copious amounts of IFNγ and TNFα that in turn affect hematopoiesis; besides, chronic (latent or active) viral infections can induce chronic inflammation, associated with increased risk of developing BM pathology. Recognition of viral PAMPs by PRRs leads to the production of type I IFNs, with antiviral and immune stimulatory properties that are beneficial for viral clearance. Short-term effects of type I IFNs on hematopoiesis point toward temporary aplasia and potential skewing to megakaryopoiesis, but the long-term effects of type I IFN signaling on hematopoiesis are still under debate and may be less deleterious than those described for IFNγ, due to the presence of regulatory mechanisms. Yet, our understanding of the full breadth by which viral infections affect hematopoiesis remains limited. It is thus important to further unravel the responsible cellular and molecular players in this process and their complex interplay in order to adequately treat or prevent anemia and BM failure in patients with viral infections.

## Author Contributions

MP and MN initiated and supervised the project, ME and MN generated the figure, MP, ME, and MN wrote the manuscript.

## Conflict of Interest Statement

The authors declare that the research was conducted in the absence of any commercial or financial relationships that could be construed as a potential conflict of interest.
